# High expression of KNL1 in prostate adenocarcinoma is associated with poor prognosis and immune infiltration

**DOI:** 10.3389/fgene.2022.1100787

**Published:** 2023-01-06

**Authors:** Yetao Zhang, Qianying Ji, Jun Wang, Yuxiang Dong, Mingyang Pang, Shengqiang Fu, Yong Wei, Qingyi Zhu

**Affiliations:** ^1^ Department of Urology, The Second Affiliated Hospital of Nanjing Medical University, Nanjing, Jiangsu, China; ^2^ Nanjing University of Chinese Medicine, Nanjing, Jiangsu, China

**Keywords:** prostate adenocarcinoma, Knl1, bioinformatics analysis, prognosis, immune infiltration

## Abstract

Prostate adenocarcinoma (PRAD) is a common malignancy with increasing morbidity and mortality. Kinetochore scaffold 1 (KNL1) has been reported to be involved in tumor progression and prognosis in other tumors, but its role in PRAD has not been reported in detail. KNL1 expression analysis, clinicopathological parameters analysis, prognostic correlation analysis, molecular interaction network and functional abdominal muscle analysis and immune infiltration analysis by using multiple online databases and downloaded expression profile. The results suggest that KNL1 is highly expressed in PRAD, which is associated with worse prognosis in PRAD patients. KnL1-related genes are highly enriched in mitotic function, which is considered to be highly related to the development of cancer. Finally, KNL1 expression is associated with a variety of tumor infiltrating immune cells, especially Treg and Th2 cells. In conclusion, our findings provide preliminary evidence that KNL1 may be an independent prognostic predictor of PRAD and is associated with immune infiltration.

## 1 Introduction

PRAD is the second most common cancer and the fifth most fatal malignancy in men worldwide (Hu et al., 2020). In China, with the aging of population and the improvement of living standards, the incidence and case fatality rate continue to increase. The 5-year survival rate of PRAD is approximately 70%–100%. However, for castration-resistant patients with distant metastases, the 5-year survival rate is only 30% (Carneiro et al., 2017). In spite of major advances in surgery, hormone deprivation therapy, and chemotherapy, the effectiveness of advanced PRAD remains limited (Lu et al., 2017; Winoker et al., 2018). Therefore, there is a need to identify additional novel diagnostic and prognostic targets for PRAD.

Tumor microenvironment (TME) is a dynamic and complex environment around tumor cells, which is composed of a variety of secreted cytokines and cells (Wang et al., 2022). Among them, the immune cells infiltrating into the tumor microenvironment are the key to play the role of tumor immunogenicity and affect the development and treatment of cancer (Hu et al., 2020). In recent years, immune-related therapies, especially acting on immune checkpoints such as programmed death-1 (PD-1)/PD-L1 and cytotoxic T-lymphocyte-associated protein 4 (CTLA-4), have led to breakthroughs in the treatment of a variety of malignancies (Abida et al., 2019; Cha et al., 2020). However, positive reactions are rarely observed in treated PRAD patients (Narayan et al., 2022). Hence identifying more immune targets or new immune mechanisms is necessary.

KNL1, also known as cancer susceptibility candidate 5 (CASC5), the protein encoded by this gene is an integral part of multiprotein assembly and is required for the generation of kinetochore/microtubule attachment and chromosome segregation (Caldas and DeLuca 2014). Thus, normal expression of KNL1 is beneficial for several aspects of mitotic progression. Previous literature has shown that dysfunctional kinetochore components can drive chromosomal instability and aneuploidy leading to tumor progression (Yuen et al., 2005; Shi et al., 2019). In recent years, the high expression of KNL1 in cancer and related cases of promoting the occurrence and development of cancers such as colon cancer (Bai et al., 2019), gastric cancer (Song et al., 2018) and lung cancer (Cui et al., 2020) have also been reported. However, it is unclear whether KNL1 in PRAD has potential function and is involved in immunity infiltration.

Here, we first identified the expression of KNL1 in PRAD, and investigated the correlation between KNL1 and clinical parameters and prognosis of PRAD. The biological function of KNL1 in PRAD was explored by mining its related genes, constructing the interaction network, and performing multi-angle functional enrichment analysis. Finally, this study revealed the relationship between KNL1 expression and tumor immune invasion.

## 2 Materials and methods

### 2.1 Download of public dataset

We downloaded gene expression profiles and clinical data from The Cancer Genome Atlas (TCGA) (https://cancergenome.nih.gov/) (Wang et al., 2016), including 499 tumor samples and 52 normal samples from PRAD patients.

### 2.2 Explore the differential expression of KNL1 in online databases

Tumor Immunity Estimation Resource (TIMER) (http://timer.cistrome.org/) database is used to identify KNL1 expression in multiple tumor types (Li et al., 2020). Then, the expression spectrum data of TCGA were used to analyze the difference in expression of paired and unpaired KNL1 samples. Correlations between KNL1 expression and PRAD molecular subtypes or immune subtypes were explored from the Tumor-Immune System Interactions Database (TISIDB) (http://cis.hku.hk/TISIDB/browse.php), which integrates multiple data types to assess tumor and immune system interactions (Ru et al., 2019). The Human Protein Atlas (HPA) website (https://www.proteinatlas.org/) was used to compare KNL1 expression in normal and tumor tissues at the protein level.

### 2.3 Cell lines and cell culture

Human normal prostate epithelial cell line RWPE-1 and human prostate cancer cell lines DU-145, 22RV1, PC-3, VCaP, and LNCaP were obtained from the Chinese Academy of Sciences Cell Bank (Shanghai, China). Cells were cultured in Dulbecco’s modified Eagle’s medium supplemented with 10% fetal bovine serum at 37 °C with 5% CO2.

### 2.4 RNA isolation and quantitative reverse Transcriptase-PCR assays

Total RNA from cells was extracted using TRIzol reagent (Life Technologies, CA, United States) following the manufacturer’s protocol. For quantitative real-time RT-PCR, cDNA synthesis was performed using 500 ng RNA per sample using RT reagent (TaKaRa, Dalian, China) according to the manufacturer’s instructions. qRT-PCR amplification was performed on a StepOnePlus real-time PCR system (Applied Biosystems, CA, United States), and data were analyzed using the 2^−ΔΔCT^ method, with GAPDH RNA as an endogenous control. The primer sequences were as follows: KNL1, forward 5′-ACC​TCT​CTG​GAC​TTC​AGC​ACT​TAC​C-3′ and reverse 5′-TCT​GTA​TCA​AGA​TGT​GGA​CCT​GGA​G-3′; GAPDH, forward 5′-ATG​GTG​AAG​GTC​GGT​GTG​AA-3′ and reverse 5′-GAG​TGG​AGT​CAT​ACT​GGA​AC-3′.

### 2.5 Correlation analysis of KNL1 expression with clinical characteristics

The relationship between KNL1 expression and clinical situation was analyzed from eight aspects using TCGA expression profile and clinical information data. In addition, logistics regression based on KNL1 differential expression is performed.

### 2.6 Survival prognosis and diagnostic value analysis

The Kaplan–Meier plotter (Liu et al., 2018) and forest map were used to assess KNL1 expression and the prognosis of cancer. Univariate and multivariate Cox analyses were used to evaluate the value of KNL1 gene as a prognostic indicator. Furthermore, the receiver operating characteristic (ROC) curve is used to assess the diagnostic value of KNL1 in PRAD.

### 2.7 Interaction network building

GeneMANIA (https://genemania.org/) (Warde-Farley et al., 2010) and STRING (https://cn.string-db.org/) (Szklarczyk et al., 2021) websites were used to construct gene-gene and protein-protein interaction networks of KNL1 to display molecules co-expressed with KNL1, and to evaluate the functions of these genes. The correlation analysis of nine KNL1-related molecules was done using TCGA expression data.

### 2.8 DEGs between KNL1 high and low expression groups in PRAD

We investigated the differences between different KNL1 expression groups based on the median KNL1 expression level in PRAD. Volcanic figure threshold for | log2 fold - change (FC) | > 1.0, after the adjustment *p* values <.05. Heat maps of the threshold for | log2 fold - change (FC) | > 2.0, the adjusted *p* values <.01. Then, we performed Gene Ontology (GO) and Kyoto Encyclopedia of Genes and Genomes (KEGG) enrichment analysis of DEGs which are upregulation (Yu et al., 2012).

### 2.9 Correlation analysis between KNL1 and tumor-infiltrating immune cells

We assessed the correlation of KNL1 expression with the abundance of six tumor-infiltrating immune cells (TIICs) by TIMER, including B-cell, CD4^+^ T-cell, CD8^+^ T-cell, neutrophils cells, macrophages and dendritic cells (DC). At the same time, we also use this database investigated the correlation between KNL1 expression and different immune cell marker genes using the correlation module, and verified them again in the Gene Expression Profiling Interaction Analysis (GEPIA2) database (http://gepia2.cancer-pku.cn/) (Tang et al., 2019). Gene Set Enrichment Analysis (GSEA) enrichment of DEGs, and the immunologic signature gene sets were selected as datasets for GSEA analysis (Subramanian et al., 2005), which are derived from Molecular Signatures Database (MSigDB) (http://www.gsea-msigdb.org/gsea/msigdb/index.jsp) (Liberzon et al., 2015). The correlation of KNL1 with the markers of 24 tumor-infiltrating immune cells were estimated. Meanwhile, we performed an analysis of the difference in TIICs in 24 between the low and high KNL1 expression groups (Bindea et al., 2013). Both of the above use the single sample GSEA (ssGSEA) algorithm (Hänzelmann et al., 2013).

### 2.10 Statistical analysis

R software (version 3.6.3) was used to process the data and plot the images. The Spearman correlation coefficient reflects the degree of correlation among different genes. The R packages and associated code used have been consolidated as raw data for submission. A value of *p* < .05 was considered statistically significant in all analyses.

## 3 Results

### 3.1 KNL1 expression in various types of human cancers

**FIGURE 1:**
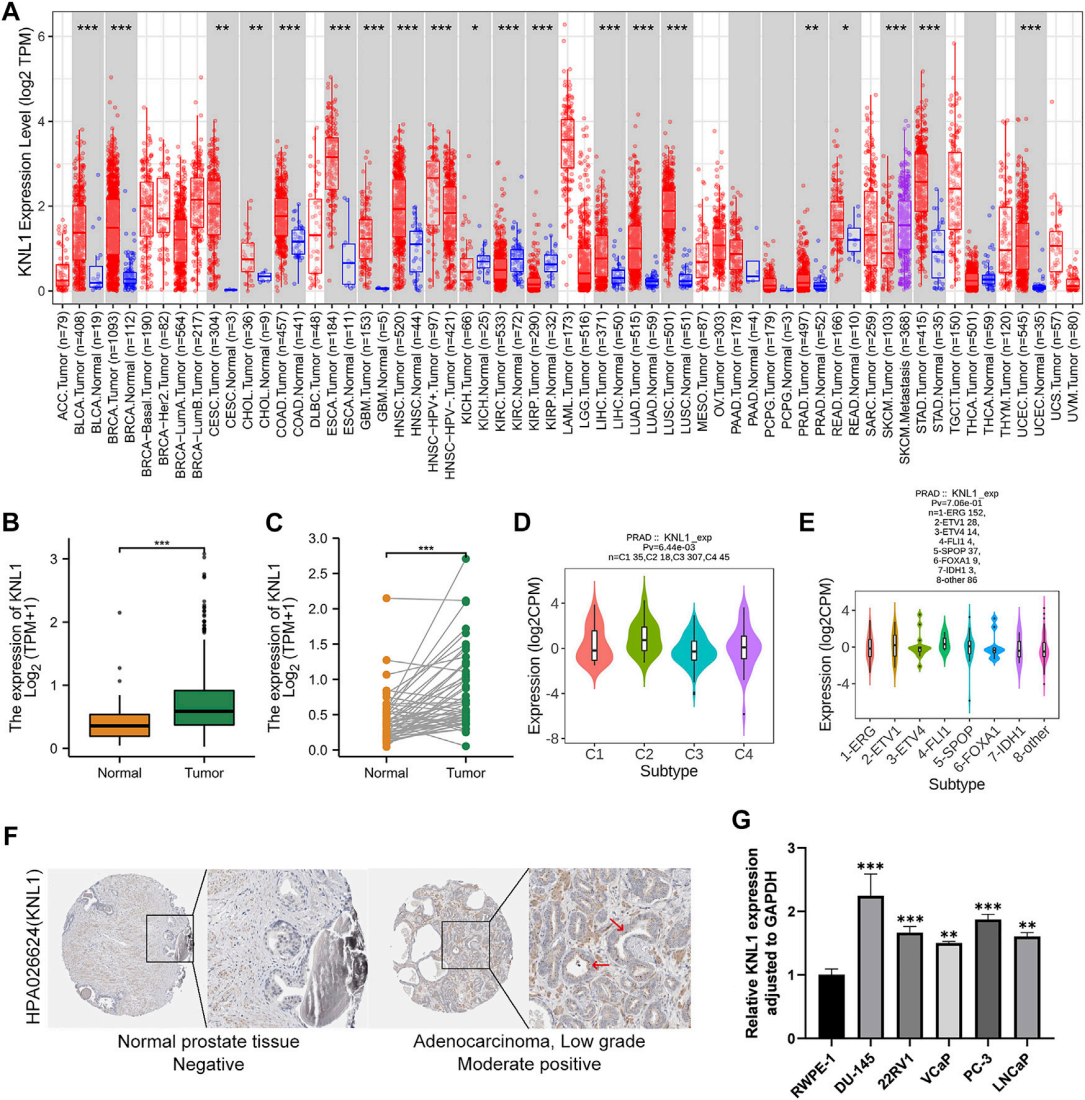
KNL1 expression levels in human cancers. **(A)** KNL1 mRNA levels in different tumor types from TCGA database were determined by TIMER. **(B)** KNL1 is expressed at higher levels in PRAD than in non-cancerous adjacent tissues. **(C)** mRNA expression levels of DRP1 in matched PRAD and adjacent non-cancerous samples in the TCGA database were compared. **(D)** Correlations between KNL1 expression and molecular subtypes in PRAD. **(E)** Correlations between KNL1 expression and immune subtypes in PRAD. **(F)** KNL1 protein levels in normal prostate and PRAD were visualized using immunohistochemistry *via* HPA. (*p < .05, **p < .01, ***p < .001). **(G)** qRT-PCR analysis of KNL1 expression in normal human prostate cell line (RWPE-1) and five prostate cancer cell lines (DU-145, 22RV1, VCaP, PC-3, LNCaP). *p < .05, **p < .01, ***p < .001.

**FIGURE 2:**
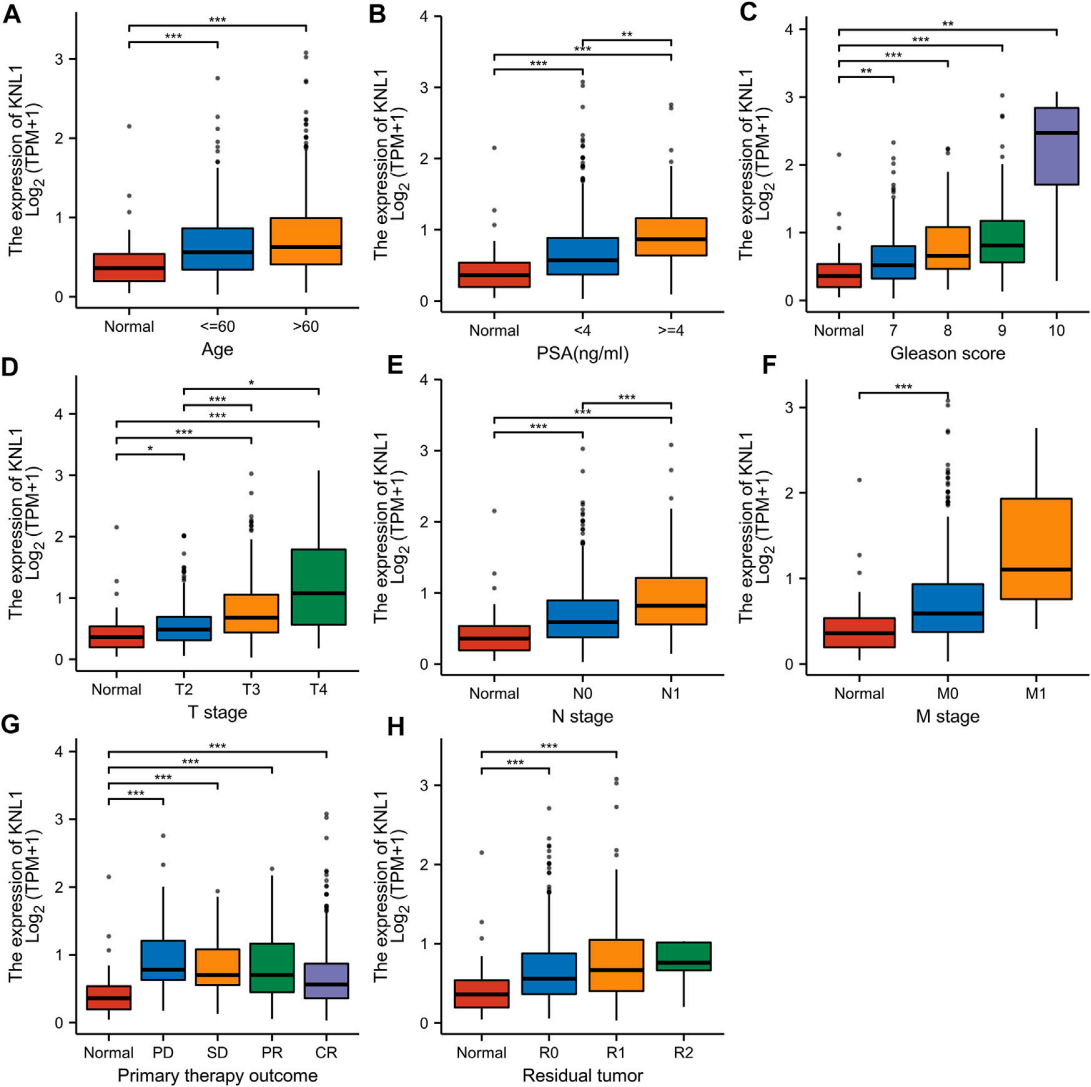
Associations between KNL1 expression and different clinical characteristics in PRAD. **(A)** Age; **(B)** PSA (ng/ml); **(C)** Gleason score; **(D)** T stage; **(E)** N grade; **(F)** M grade. **(G)** Primary therapy outcome; **(H)** Residual tumor (*p < .05, **p < .01, ***p < .001).

The TIMER database findings indicated that KNL1 gene is differentially expressed in a variety of cancers. And compared with normal tissues, the expression level in tumor tissues of PRAD patients was higher (*p* < .01) (Figure 1A Figure 2). Subsequently, we analyzed the expression profile data downloaded from TCGA-PRAD, and it could be seen that KNL1 gene was relatively highly expressed in tumor tissues, both in unpaired (*p* < .001) and paired (*p* < .001) samples analysis (Figures 1B,C). By investigating TISIDB, we found that KNL1 was expressed differently in different immune subtypes of PRAD (C1: wound healing, C2: IFN-gamma dominant, C3: inflammatory, C4: lymphocyte depleted) (Figure 1D), but its expression has no correlation with different molecular subtypes (Figure 1E). Then, immunohistochemical analysis of the HPA database showed that the KNL1 protein content was also increased in PRAD (Figure 1F). Furthermore, qRT-PCR assay showed that KNL1 was highly expressed in five prostate cancer cell lines compared with normal cells (Figure 1G).

### 3.2 Relationship between KNL1 expression and clinicopathological parameters

Given the high expression of KNL1 gene in PRAD, we further explored the relationship between KNL1 expression and clinical case parameters of PRAD patients. As for the division of age groups, a number of studies have shown that in recent years, the morbidity and mortality of the age group over 60 have shown exponential growth, and the growth rate is much higher than that of the relatively young age group, so they are divided into two groups: ≤60 and >60 (Liu et al., 2019). At the same time, the positive critical value of 4.0 ng/ml with high sensitivity was selected for grouping based on important evidence such as the setting of The Prostate, Lung, Colorectal and Ovarian (PLCO) Cancer Screening Trial in the United States (Andriole et al., 2009; Lavallée et al., 2016). After sorting and analyzing the expression profile data and clinicopathological parameter files in TCGA-PRAD using R software, we observed the mRNA level of KNL1 was related to PSA (ng/ml), Gleason score, tumor size, regional lymph node metastasis. However, KNL1 expression was not correlated with age, distant metastasis, primary therapy outcome and residual tumor (Figure 2). Logistic regression indicated that the expression of KNL1 in T3 & T4 overtopped T2 (*p* < .001), positive lymph node metastasis is more than negative (*p* = .001), stable disease (SD) & progressive disease (PD) overtopped partial response (PR) & complete response (CR) (*p* < .001), PSA≥4 ng/ml overtopped PSA<4 ng/ml (*p* = .002), and high Gleason score (8&9&10) overtopped medium Gleason score (6&7) (*p* < .001). There was no difference in age (*p* = .194), distant metastasis (*p* = .590) and residual tumor (*p* = .062) (Table 1).

**TABLE 1:** Association between KNL1 expression and clinicopathologic parameters by Logistic regression.

Characteristics	Total(N)	Odds Ratio (95%CI)	*p*-Value
Age (>60 vs≤60)	499	1.264 (0.888–1.802)	0.194
T stage (T3&T4 vs T2)	492	2.242 (1.549–3.263)	<0.001
N stage (N1 vs N0)	426	2.421 (1.443–4.175)	0.001
M stage (M1 vs M0)	458	1.939 (0.185–41.906)	0.590
Primary therapy (SD&PD vs PR&CR)	438	2.861 (1.581–5.414)	<0.001
Residual tumor (R1&R2 vs R0)	468	1.448 (0.983–2.140)	0.062
PSA (ng/ml) (≥4 vs <4)	442	4.916 (1.972–14.901)	0.002
Gleason score (8&9&10 vs 6&7)	499	3.280 (2.264–4.786)	<0.001

### 3.3 Prognostic potential of KNL1 expression in PRAD

**FIGURE 3:**
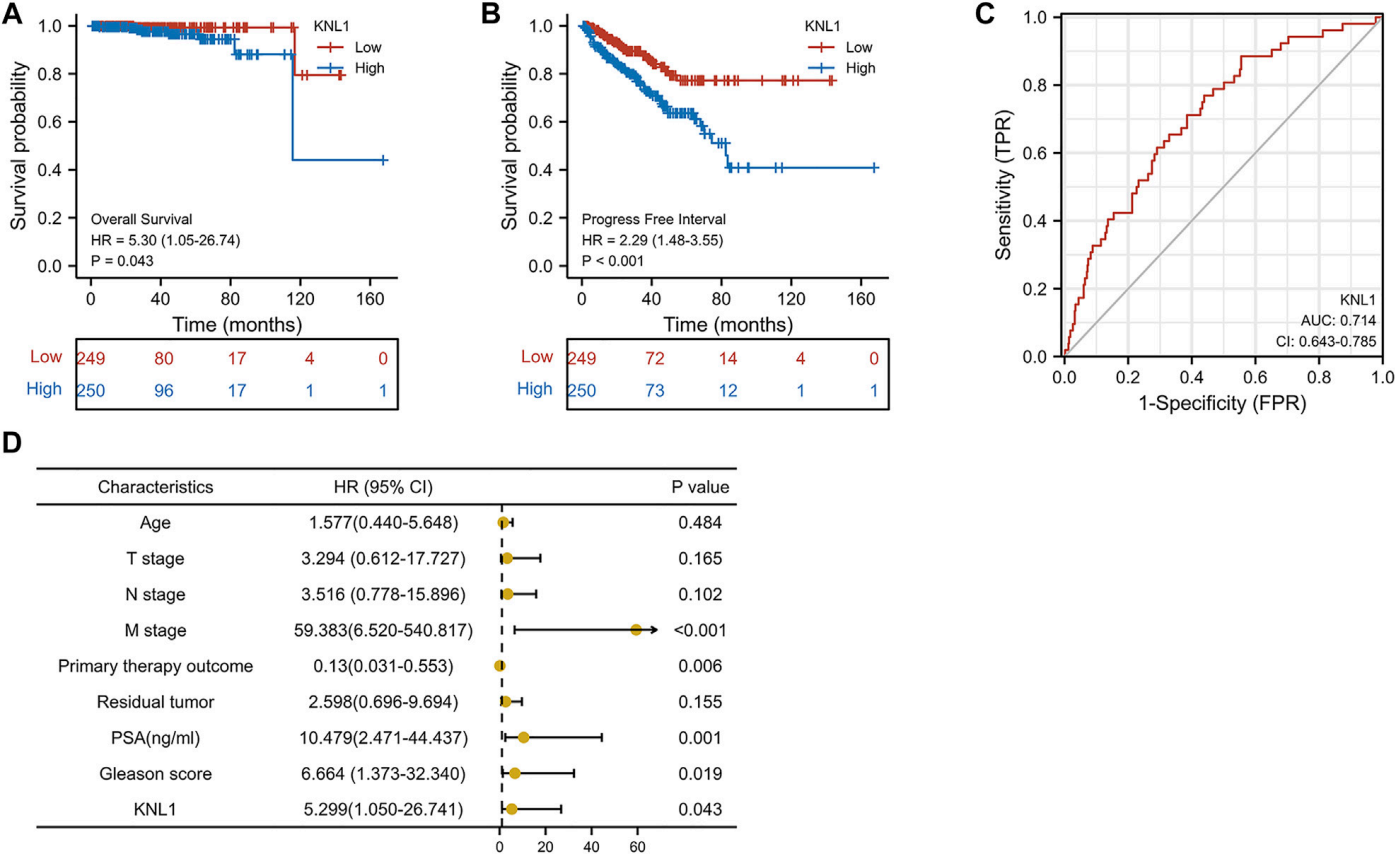
Correlation between KNL1 and the prognosis. KNL1 expression was significantly negatively correlated with OS **(A)** and PFI **(B)** in TCGA. **(C)** Receiver operating characteristic (ROC) curve for KNL1 expression in PRAD. **(D)** Forest map of KNL1 expression and other clinicopathological parameters.

To investigate the relationship between KNL1 expression and prognosis of PRAD patients, we conducted a comprehensive analysis of expression profile and survival data in TCGA-PRAD. Prognostic survival analysis showed that KNL1 expression was negatively correlated with overall survival (OS) (HR = 5.30, *p* = .043) and progress free interval (PFI) (HR = 2.29, *p* < .001) (Figures 3A,B). Receiver operating characteristic curve showed that KNL1 had a certain accuracy (AUC = .714) in predicting PRAD (Figure 3C). Finally, we illustrated the relationship between KNL1 expression and other clinicopathological parameters and OS using COX analysis. The univariate Cox analysis showed that distant metastasis (HR = 59.383, *p* < .001), Primary therapy outcome (HR = .130, *p* < .001), PSA level (HR10.479, *p* = .001), Gleason score (HR = 6.664, *p* = .019) and KNL1 expression (HR = 5.299, *p* = .043) were associated with OS. The multivariate analysis indicated that distant metastasis (HR = 63.927, *p* = .007) had independent prognostic value (Table 2). The forest map (Figure 3D) depicts the results of the univariate analysis.

**TABLE 2:** Univariate and multivariate Cox analysis of clinicopathological parameters and OS in patients with PRAD.

Characteristics	Univariate analysis		Multivariate analysis	
	HR (95% CI)	*p* value	HR (95% CI)	*p* value
Age	1.577	.484		
	(.440−5.648)			
T stage	3.294	.165		
	(.612−17.727)			
N stage	3.516	.102		
	(.778−15.896)			
M stage	59.383	<.001	63.927	.007
	(6.520−540.817)		(3.138−1302.324)	
Primary therapy outcome	.130	.006	.325	.200
	(.031−.553)		(.058−1.815)	
Residual tumor	2.598	.155		
	(.696−9.694)			
PSA(ng/ml)	10.479	.001	2.896	.268
	(2.471−44.437)		(.442−18.974)	
Gleason score	6.664	.019	2.118	.447
	(1.373−32.340)		(.307−14.623)	
KNL1	5.299	.043	2.723	.290
	(1.050−26.741)		(.427−17.373)	

#### 3.3.1 Construction of interaction network of KNL1 and KNL1-correlated genes

**FIGURE 4:**
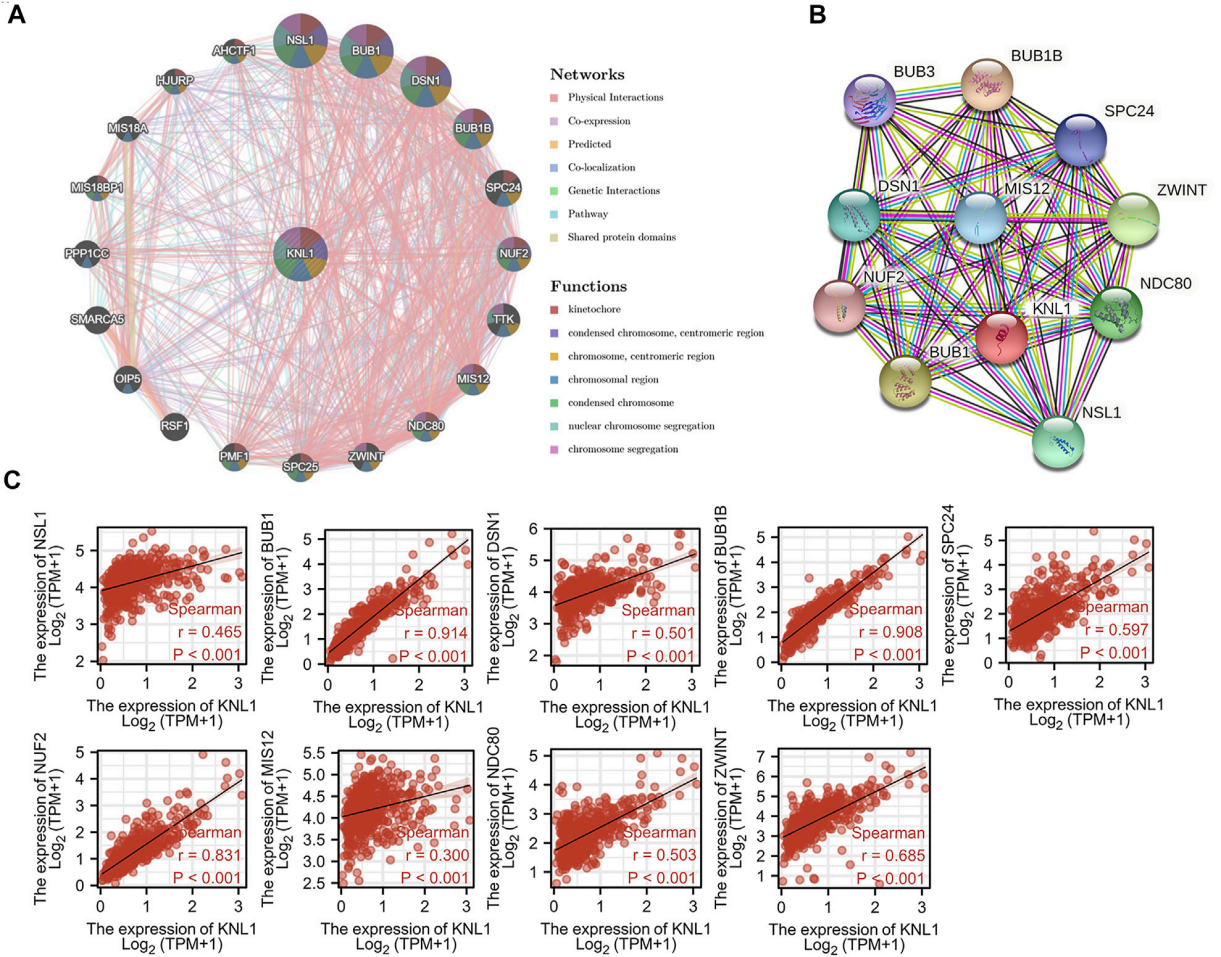
Co-expression genes of KNL1. **(A)** Network of the top 20 genes associated with KNL1 in GeneMANIA. **(B)** STRING. **(C)** Correlation analysis of KNL1 and its co-expressed genes.

To explore the mechanism of KNL1 in PRAD, we constructed a gene-gene interaction network for KNL1 using the GeneMANIA database, and analyzed the functions of these genes. KNL1 is surrounded by 20 gene nodes, which represent genes significantly associated with KNL1 (Figure 4A). Subsequent functional analysis revealed that the genes encoded proteins associated with the following terms: kinetochore, chromosomal region, condensed chromosome, chromosome (centromeric region), condensed chromosome (centromeric region), chromosome segregation and nuclear chromosome segregation (Figure 4A). At the same time, the KNL1-related molecular network at the protein level was constructed using the STRING database. We show here the PPI network formed by the top 10 KNL1-related molecules, containing 11 nodes and 54 edges, with an average local clustering coefficient of .982 (Figure 4B). Taking the intersection of molecules contained in the above two networks, the following molecules can be obtained: NSL1, BUB1, DSN1, BUB1B, SPC25, NUF2, MIS12, NDC80, ZWINT. The correlation analysis between their expression and KNL1 expression in PRAD was performed using TCGA-PRAD expression profile (Figure 4C).

### 3.4 Functional enrichment analyses of KNL1 and co-expressed genes

**FIGURE 5:**
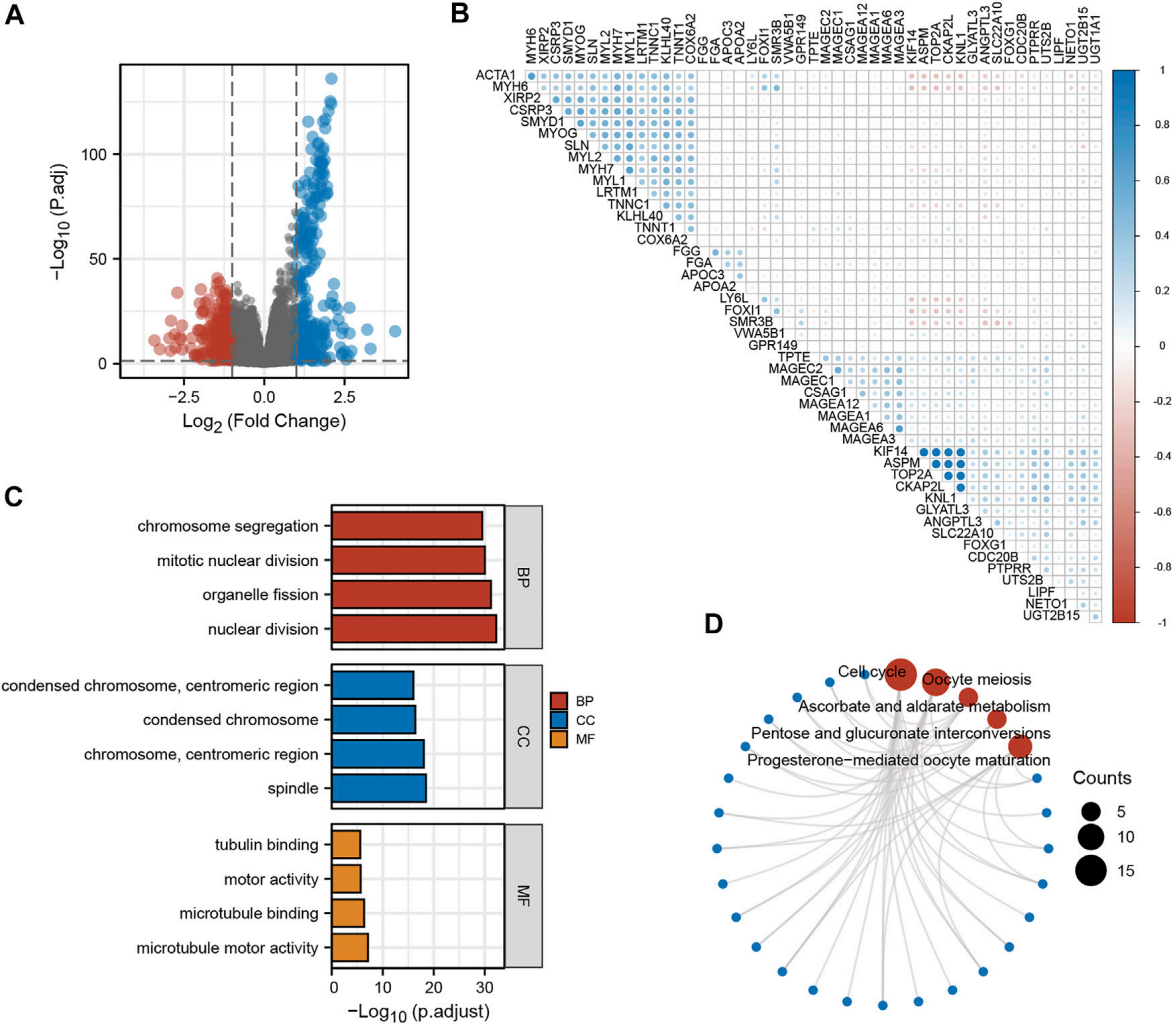
Screening and functional enrichment analysis of related genes. **(A)** Volcano plot for single-gene difference analysis (|LogFC|>1, p. adj<.05). **(B)** Correlation Heatmap for Single Gene Difference Analysis (|LogFC|>2, p. adj<.01). GO **(C)** and KEGG **(D)** analysis of 300 genes positively correlated with KNL1.

To further understand the role of KNL1 in PRAD, the expression profile data of TCGA-PRAD were collated and analyzed as follows. Firstly, the differential analysis between the high and low expression groups of KNL1 was carried out to obtain the differential genes related to KNL1. The volcano plot shows the case Based on the criteria |LogFC|>1 and p. adj<.05 (Figure 5A). Co-expression heat map shows the correlation of differential genes with KNL1 when the threshold is set to |LogFC|>2 and p. adj<.01 (Figure 5B). Subsequently, 338 up-regulated differential genes were included for GO and KEGG enrichment analysis. The top four enriched biological process (BP) terms were nuclear division, organelle fission, mitotic nuclear division, and chromosome segregation (Figure 5C). The following cellular component (CC) terms were significantly correlated with KNL1: spindle, chromosome (centromeric region), condensed chromosome, and condensed chromosome (centromeric region) (Figure 5C). Molecular function (MF) terms showed that KNL1 was significantly correlated with the microtubule motor activity, microtubule binding, motor activity, and tubulin binding (Figure 5C). In the KEGG analysis, these genes were significantly enriched in Cell cycle, Oocyte meiosis, Ascorbate and aldarate metabolism, Pentose and glucuronate interconversions, and Progesterone-mediated oocyte maturation (Figure 5D).

### 3.5 Relationship between KNL1 expression and TIICs

**FIGURE 6:**
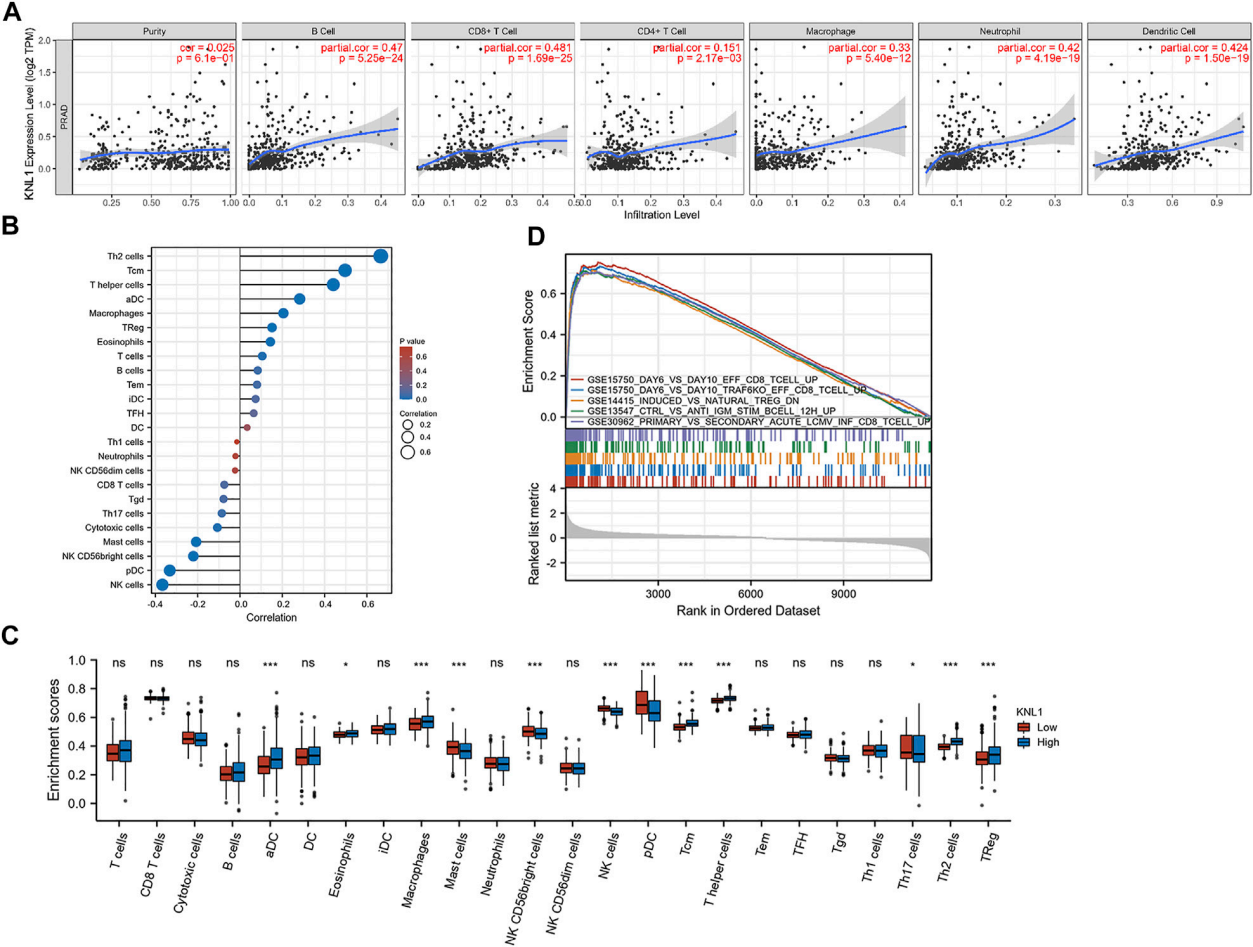
Correlation between KNL1 expression and TIICs. **(A)** Correlation between KNL1 expression and six types of immune cells in the TIMER database. **(B)** Lollipop graphs of correlation between KNL1 and biomarkers of 24 immune cells. **(C)** The distribution of 24 subtypes of immune cells in low and high KNL1 expression group. **(D)** GSEA analysis of differential genes in immune-related datasets by single-gene analysis. (Ns: no significance; *p < .05; **p < .01; ***p < .001).

Through the analysis of the immune module of TIMER database, we found that KNL1 was positively correlated with the following six kind of TIICs: B-cell (r = .47, *p* = 5.25e-24), CD8 + T-cell (r = .481, *p* = 1.69e-25), CD4 + T-cell (r = .151, *p* = 2.17e-03), macrophages (r = .33, *p* = 5.40e-12), neutrophils (r = .42, *p* = 4.19e-19) and DC (r = .424, *p* = 1.50e-19) (Figure 6A). We then used the ssGSEA algorithm to infer the infiltration of immune cells 24 in TCGA-PRAD samples. The expression of 8 markers had positive correlations with KNL1 expression in PRAD: Th2 cells (r = .666, *p* < .001), T central memory (Tcm) (r = .497, *p* < .001), T helper cells (r = .441, *p* < .001), activated DC (aDC) (r = .282, *p* < .001), Macrophages (r = .205, *p* < .001), Treg (r = .152, *p* < .001), Eosinophils (r = .144, *p* = .001), T-cell (r = .105, *p* = .019) (Figure 6B). Meanwhile, NK cells (r = -.367, *p* < .001), Plasmacytoid DC (pDC) (r = -.332, *p* < .001), NK CD56bright cells (r = -.221, *p* < .001), Mast cells (r = -.207, *p* < .001), and cytotoxic cells (r = -.107, *p* = .017) expression was negatively associated with KNL1 expression in PRAD (Figure 6B). In addition, we compared the differences of 28 TIICs between KNL1 high expression group and KNL1 low expression group. The result indicated that the high-expression group had more aDC (*p* < .001), Eosinophils (*p* < .05), Macrophages (*p* < .001), Tcm (*p* < .001), T helper cells (*p* < .001), Th2 cells (*p* < .001) and Tregs (*p* < .001) (Figure 6D). Moreover, we performed immune-related GSEA analysis using the differential genes between the two groups, and the results showed that the top five gene sets were associated with B-cell, CD8 + T-cell, and Tregs (Figure 6C).

### 3.6 Correlation between KNL1 expression and TIICs marker gene

The correlation between KNL1 expression and TIICs marker genes was studied by TIMER. After purity adjustment, KNL1 was positively correlated with marker genes of all T-cell (general), monocyte, TAM, M1 macrophages, M2 macrophages, dendritic cells, Th1 cells, T follicular helper (Tfh) cells and Treg cells. KNL1 expression was positively correlated with some marker genes of CD8 + T-cell, B-cell, neutrophil, NK cells, Th2 cells, Th17 cells, and T-cell exhaustion (Table 3). The correlation between some TIICs marker genes and KNL1 expression was explored by using GEPIA2 database. In tumor tissues, KNL1 was positively correlated with the marker genes of TAM, M2 macrophages and Tregs (Table 4).

**TABLE 3:** Correlation analysis between KNL1 and gene markers of immune cells in TIMER.

Description	Marker genes	None cor	P	Purity cor	P
CD8 + T-cell	CD8A	0.227	***	0.262	***
	CD8B	0.101	0.070	0.097	0.120
T-cell (general)	CD3D	0.115	*	0.123	*
	CD3E	0.198	***	0.224	***
	CD2	0.243	***	0.250	***
B-cell	CD19	0.074	0.219	0.063	0.362
	CD79A	0.169	**	0.165	**
	CD27	0.163	**	0.180	**
Monocyte	CD14	0.201	***	0.216	***
	CD86	0.448	***	0.466	***
	CD115 (CSF1R)	0.387	***	0.405	***
TAM	CD68	0.471	***	0.485	***
	IL10	0.386	***	0.376	***
M1 Macrophage	INOS (NOS2)	0.153	**	0.114	*
	IRF5	0.476	***	0.544	***
	COX2(PTGS2)	0.210	***	0.193	***
M2 Macrophage	VSIG4	0.439	***	0.451	***
	MS4A4A	0.419	***	0.447	***
Neutrophil	CD66b (CEACAM8)	0.043	0.443	0.056	0.359
	CD11b (ITGAM)	0.397	***	0.416	***
	CCR7	0.267	***	0.293	***
Natural killer cell	KIR2DL1	0.061	0.345	0.052	0.485
	KIR2DL3	0.071	0.263	0.096	0.140
	KIR2DL4	0.149	**	0.162	**
	KIR3DL1	0.150	**	0.156	**
	KIR3DL2	0.070	0.269	0.053	0.478
	KIR3DL3	0.041	0.544	0.023	0.803
	KIR2DS4	0.046	0.503	0.041	0.601
Dendritic cell	BDCA-1 (CD1C)	0.286	***	0.297	***
	BDCA-4 (NRP1)	0.520	***	0.509	***
	CD11c (ITGAX)	0.396	***	0.443	***
Th1	T-bet (TBX21)	0.219	***	0.269	***
	STAT4	0.246	***	0.259	***
	IFN-γ (IFNG)	0.281	***	0.286	***
	TNF-α (TNF)	0.244	***	0.595	***
	STAT1	0.591	***	0.222	***
Th2	GATA3	0.035	0.523	0.004	0.955
	STAT5A	0.175	***	0.186	***
	STAT6	0.198	***	0.198	***
Tfh	BCL6	0.196	***	0.242	***
	IL21	0.172	***	0.168	**
Th17	STAT3	0.528	***	0.521	***
	IL17A	0.109	*	0.038	0.529
Treg	FOXP3	0.442	***	0.490	***
	CCR8	0.540	***	0.547	***
	TGFβ (TGFB1)	0.223	***	0.288	***
	STAT5B	0.411	***	0.427	***
T-cell exhaustion	PD-1 (PDCD1)	0.097	0.082	0.152	**
	CTLA4	0.189	***	0.217	***
	LAG3	-0.123	*	-0.124	*
	TIM-3 (HAVCR2)	0.428	***	0.453	***
	GZMB	0.113	*	0.114	0.055

TAM, tumor-associated macrophage; Th, T helper cell; Tfh, Follicular helper T-cell; Treg, regulatory T-cell; Cor, R value of Spearman’s correlation; None, correlation without adjustment. Purity, correlation adjusted by purity.

*p < .05.

**p < .01.

***p < .001.

**TABLE 4:** Correlation analysis between KNL1 and gene markers of immune cells in GEPIA2.

Description	Marker genes	Tumor cor	P	Normal cor	P
CD8 + T-cell	CD8A	0.14	**	0.28	*
	CD8B	0.06	0.21	0.19	0.17
T-cell (general)	CD3D	0.07	0.13	0.32	*
	CD3E	0.14	**	0.35	*
	CD2	0.17	***	0.35	*
B-cell	CD19	0.05	0.26	0.23	0.11
	CD79A	0.14	***	0.51	***
	CD27	0.12	**	0.38	**
TAM	CD68	0.43	***	0.46	***
	IL10	0.32	***	0.33	*
M1 Macrophage	INOS (NOS2)	0.06	0.18	0.19	0.18
	IRF5	0.43	***	0.08	0.57
	COX2 (PTGS2)	0.10	*	0.08	0.56
M2 Macrophage	VSIG4	0.33	***	0.34	*
	MS4A4A	0.36	***	0.45	***
Treg	FOXP3	0.31	***	0.41	**
	CCR8	0.39	***	0.47	***
	TGFβ (TGFB1)	0.13	**	0.18	0.21
	STAT5B	0.20	***	0.16	0.25

TAM, tumor-associated macrophage; Treg, regulatory T-cell; Cor, R value of Spearman’s correlation.

*p < .05.

**p < .01.

***p < .001.

## 4 Discussion

PRAD is a complex but common malignancy that causes about 1.3 million new cases and 360,000 deaths worldwide each year. It has become one of the most common urogenital malignancies in elderly Chinese men (Li et al., 2021). KNL1 is an important regulatory gene in mitosis (Krenn et al., 2012). It integrates the functions of various mitotic regulators, including BUB1 and BUBR1, and is the Kinetochore component required for the spindle assembly checkpoint (SAC), which protects the correct segregation of chromosomes during mitosis. Defects in KNL1 function have been associated with genomic instability, leukemia, microcephaly, and neurological disorders (Shi et al., 2019). Previous literature has pointed out that almost all solid tumors exhibit genomic instability at the chromosomal level. Strong experimental evidence supports that chromosomal instability phenotypes occur early in cancer development and represent an important step in tumor progression (Shih et al., 2001). Recent reports suggest that the long-term proliferation of aneuploid cancer cells is threatened by SAC inhibition (Cohen-Sharir et al., 2021). Jennifer G. put forward that KNL1 may be a platform for SAC-activation and SAC-silencing proteins (Caldas and DeLuca 2014). In recent years, more and more studies have shown that KNL1 dysregulation may lead to the progression of colorectal cancer (Bai et al., 2019) and gastric cancer (Song et al., 2018). Down-regulation of KNL1 can inhibit the growth and induce cell death of cervical cancer and breast cancer cells (Urata et al., 2015). Therefore, KN1L is indeed linked to a variety of solid tumors. However, no relevant studies have been found on KNL1 in PRAD, and whether KNL1 is related to immune infiltration in PRAD is still unclear.

Through database mining, this study found that KNL1 was highly expressed in PRAD tissues compared with normal tissues. This was confirmed by our qPCR assay. Clinical correlation analysis showed that KNL1 expression was associated with PSA level, Gleason score, tumor size, regional lymph node metastasis. However, no difference was observed in age, distant metastasis, primary therapy outcome and residual tumor, which may be attributed to the lack of a large number of clinical data. In addition, multivariate regression analysis showed that there was a causal relationship between distant metastasis and prognosis to some extent, but there was no difference in correlation analysis, which we believed was related to incomplete clinical data and large differences in sample size between groups. If more clinical data can be added, more stable results may be obtained. Prognostic analysis showed that high expression of KNL1 was associated with poor prognosis. These implications suggest that high KNL1 expression is associated with PRAD progression and may be a potential independent predictor.

The gene network construction and functional enrichment analysis showed that the expression of KNL1 and its related genes is highly correlated with mitosis and cell cycle, and they are highly enriched in kinetochore, chromosomal region, chromosome segregation and other biological functions. At the same time, we can see, in the database identified molecules that are associated with KNL1 height mostly for SAC related gene (BUB1 BUB1R, SPC25, MIS12, NDC80, ZWINT), The highly related genes co-up-regulated with KNL1 in PRAD samples (KIF14, ASPM, CKAP2L) are also involved in spindle assembly regulation and microtubule formation, and their high expression has been reported to be associated with the occurrence and development of a variety of cancers (Pai et al., 2019; Yang et al., 2019; Monteverde et al., 2021). As mentioned above, the function of SAC is closely related to the occurrence and development of solid tumors. The results further indicated that KNL1 played an important role in the development of PRAD. Interestingly, these KNL1-related molecules (e.g., BUB1, ASPM, TOP2A) have been implicated in immune infiltration in papillary renal cell carcinoma in another report (Deng et al., 2021).

At present, the main analysis of TIICs in tumors usually focuses on T-cell, especially the related studies of CTLA-4 inhibitors and PD-1/PD-L1 inhibitors (Rowshanravan et al., 2018; Rotte 2019). At the same time, more and more researchers have paid attention to the role of B-cell and tertiary lymphoid structures in immunotherapy (Cabrita et al., 2020; Helmink et al., 2020; Fridman et al., 2022). GSEA analysis in this study showed that the gene groups co-upregulated with KNL1 were mainly enriched in CD8+T-cell, B-cell and Tregs. Our exploration of TIMER database suggests that KNL1 expression is well correlated with a variety of TIICs and markers, especially in TAM, M2 macrophages and Tregs. In addition, the GEPIA2 database analysis was used to compare KNL1 in PRAD with normal tissues and TIICs. It can be seen that the correlation between M2 macrophages and Tregs and KNL1 in tumor tissues is stronger than that in normal tissues. It is worth mentioning that the increased infiltration of these immune cells (such as Treg, Th2 cells, M2 Macrophage) may produce a worse prognosis (Ruffell et al., 2010; Mehla and Singh 2019; Tanaka and Sakaguchi 2019; Protti and De Monte 2020; Amoozgar et al., 2021). Our group analysis results exactly showed that higher expression of KNL1 brought more Th2 and Treg infiltration, and at the same time, NK cell and other anti-tumor components showed a lower enrichment level. Our findings suggest that KNL1 does have a certain effect on the immune infiltration of PRAD, and more basic experiments may better prove this view.

Although this study has improved our understanding of the correlation between KNL1 and PRAD, at the same time, there are some limitations. First of all, we mainly conducted the analysis through bioinformatics methods, and more experiments are needed to explore and verify the molecular mechanisms and biological functions related to KNL1. Secondly, both K-M plotter and ROC curve performed well in the prognostic analysis, but no good difference was observed in univariate and multivariate regression, which was attributed to the lack of a large amount of data. Therefore, we should establish our own clinical case database to expand the sample size.

## 5 Conclusion

In conclusion, the expression of KNL1 in PRAD is up-regulated, which is significantly correlated with the clinical characteristics of PRAD patients and predicts poor prognosis. This gene can be considered as an early diagnostic and independent prognostic indicator for PRAD patients. In addition, our analysis demonstrated a significant correlation between KNL1 expression and the degree of immune cell infiltration. Therefore, KNL1 may play a potentially important role in immunotherapy.

## Data Availability

The datasets presented in this study can be found in online repositories. The names of the repository/repositories and accession number(s) can be found in the article/Supplementary Material.
